# Emergence of a barium metal-organic framework for mitigating off-target effects of alpha radionuclide therapy

**DOI:** 10.7150/thno.121316

**Published:** 2026-01-01

**Authors:** Long Qiu, Jie Lyu, Yuqi Guo, Shilong Shi, Xijian Chen, Junshan Geng, Qian Xiao, Jiali Liao, Yuanyou Yang, Jinsong Zhang, Ning Liu, Feize Li

**Affiliations:** 1 Key Laboratory of Radiation Physics and Technology of the Ministry of Education, Institute of Nuclear Science and Technology, Sichuan University, Chengdu 610064, P. R. China.; 2 Department of Nuclear Medicine, Chongqing University Cancer Hospital, Chongqing 400030, P. R. China.; 3 Sichuan Engineering Research Center for Radioactive Isotope, National Engineering Research Center for Isotopes and Pharmaceuticals, Nuclear Power Institute of China, Chengdu 610005, P. R. China.; 4 Department of Nuclear Medicine, West China Hospital of Sichuan University, Chengdu, Sichuan 610041, P. R. China.

**Keywords:** ^224^Ra, MOFs, cancer therapy, nanomedicine, radiopharmaceutical

## Abstract

**Background:** ^224^Ra, an alpha-emitting radionuclide with a half-life of 3.63 d, holds significant promise in cancer therapy. However, like many other medical alpha-emitters, the development of ^224^Ra radiopharmaceuticals has long been impeded by dosimetry limitation caused by the off-target toxicity, which is tightly related to the secondary radioactivity biodistribution.

**Methods:** In this work, we propose leveraging radionuclide trap preorganized in nanoscale barium-based metal-organic framework (AEMOF-6) to overcome the off-target effects of ^224^Ra therapy. Functional side chains with high binding affinity towards ^224^Ra and its decay daughters were preinstalled inside the cavity of nanoscale AEMOF-6, constructing radionuclide trap capable of inhibiting the radioactivity leaking effectively.

**Results:** The ^224^Ra-labeled radiopharmaceutical ^224^Ra-AEMOF-6@CS demonstrates effective *in vivo* radioactivity localization ability, significant antitumor efficacy, and favorable biosafety. It was obtained with a radiochemical yield of 92.87% and a radiochemical purity of 94.75%, maintaining over 87% *in vitro* stability throughout the observation period. Integrated micro-PET/CT and micro-SPECT/CT imaging, complemented by biodistribution analyses, validated the robust stability and radioactivity localization capability of the AEMOF-6@CS nanocarrier *in vivo*. A dose-dependent antitumor effect accompanied by excellent biosafety was observed, achieving complete tumor eradication in 20%, 40%, and 60% of mice at 36 d after injection of 18.5, 37.0, and 55.5 kBq of ^224^Ra-AEMOF-6@CS, respectively.

**Conclusion:** This discovery provides a potential approach to address the challenges of radioactivity migration of ^224^Ra radiopharmaceuticals *via* radionuclide trap preorganized in nanoscale MOFs, which can also be beneficial to other alpha-emitting radiopharmaceuticals.

## Introduction

Targeted alpha (α)-therapy (TAT) has garnered sharply increasing interest in cancer therapy, also facing huge challenges from the off-target effect of the recoiling daughters of the α-emitting cytotoxin. High lethality and appropriate penetration range of α particles enable TAT to destroy tumor cells effectively and diminish unnecessary irradiation damage to adjacent normal tissues 1-4. Accordingly, TAT is becoming a vital therapeutic modularity of disseminated and metastatic cancers. Since the approval of Xofigo® ([^223^Ra]RaCl_2_) by the U.S. FDA in 2013 5, ^223^Ra-labeled TAT drugs have garnered significant attention and demonstrated excellent tumor treatment capability 6-10. On the other hand, the daughter radionuclides within the decay chain of common medical α-emitter can obtain enough recoil energy (≥ 100 keV) much stronger than the bond energy of any chemical compound (2~10 eV) 11. Consequently, the decay daughters always recoil from the targeting vector and redistribute *in vivo*, causing severe irradiation-related toxicity to patients 12-15. One of the most representative cases is ^224^Ra 16, which has been regarded as a potential medical α-emitter since 1913 17 and was once clinically applied to treat arthritis patients with ankylosing spondylitis in Germany 18. However, ^224^Ra therapy was officially withdrawn in 2005, which is just due to its significantly increased risk of renal cancer and leukemia associated with the off-target toxicity. Besides, ^223^Ra- and ^225^Ac-radiolabeled compounds have demonstrated excellent prospects in clinical practice, but their wide applications are still clouded by the significant leakage of toxic daughter radionuclides to normal organs/tissues 19-25. Obviously, how to localize the radioactivity of α-emitting radiopharmaceuticals is decisive to move TAT from bench to bedside 20-21, 26.

Nanoencapsulation is a potential approach to localizing the recoiling daughters of medical α-emitters yet requires more rational design. It has been demonstrated that some nanocarriers such as TiO_2_, Fe_3_O_4_, MOFs, NaA zeolites, nHA, and CaCO_3_ *etc.* could improve the radioactivity biodistribution of the delivered α radionuclides 26-32. Generally, medical α-radionuclides are usually required to be anchored inside these targeting vectors with multilayered coatings to achieve acceptable decay daughter localization. Complicated surface modification is extremely unfavorable for drug synthesis while the resultant radioactivity encapsulation remains limited. For instance, to effectively retain ^225^Ac and its daughters within gold nanocarriers, four layers of gadolinium phosphate must apply. A reduction by just a single layer would cause the release of more than 30% of the radioactive daughter ^213^Bi from the nanoscale radionuclide delivery vehicles 33. Furthermore, Ján Kozempel et al. 34 have found that *in vitro* tests performed under static conditions may yield false positive results while significantly higher recoil releases can be observed *in vivo* testing or a dynamic system model. This discrepancy may be caused by the *in vitro* secondary resorption of daughter radionuclides by surrounding nanoparticles. In the case employing nanoscale barium ferrite to encapsulate ^223^Ra and its progeny, a synthesis process lasting six hours was adopted but corresponding nanomedicine still exhibited rapid *in vivo* release of approximately 15%^ 211^Bi and 27% ^211^Pb, which are known as two toxic decay daughters during ^223^Ra radiotherapy 35. All these findings highlight the urgent need for the development of structurally simpler yet more efficient nanocarriers capable of sequestering the daughter radionuclides of α-cytotoxin, which is critical for advancing the broader application of TAT.

**Scheme 1:**
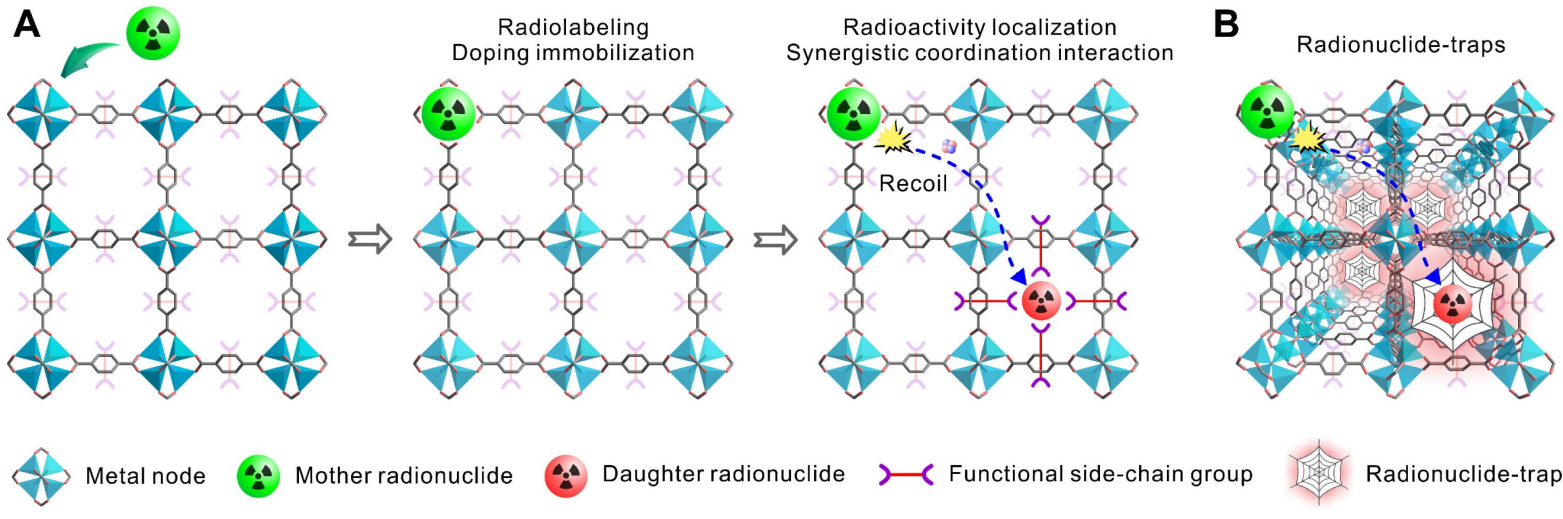
(A) Schematic illustration of metal nodes in MOFs accommodating radionuclides through metal doping, while functional side-chain groups establish strong synergistic coordination with recoiling radioactive cations. (B) The ordered and repeating pattern make MOFs able to form radionuclide traps available for capturing any recoiling daughters from the mother α-radionuclide.

In this work, we proposed a nanoencapsulation strategy applying radionuclide trap preorganized inside the cavity of an alkaline earth metal-organic framework (AEMOF-6) based on barium to localize the radioactivity of ^224^Ra and its decay daughter 36. This strategy differs from previously reported “recoil spread mitigation by nanoconstruct size/material” or “recoil spread mitigation by the nanoconstructs number/depot” 11 and is expected to achieve better radioactivity confinement of the decay daughters of ^224^Ra. There are several motivations for this design. First, ^224^Ra is a typical medical α-emitter of which the clinical application has been aborted due to the off-target effects of toxic recoiling daughters, a rational proposal able to overcome the radioactivity release is significant for not only the development of radium therapy but also other TAT formulations. Second, metal-organic frameworks (MOFs) are a range of inorganic-organic hybrid materials showing great promising in biomedicine field including radiopharmaceuticals 37-40. The metal nodes of MOFs are intrinsically excellent sites to accommodate radionuclides by metal doping while functional side-chain groups from adjacent organic linkers inside the cavities can produce strong synergistic coordination interactions towards the recoiling radioactive cations (Scheme 1A). Furthermore, the ordered and repeating pattern make MOFs able to form radionuclide traps available for capturing any recoiling daughters from the mother α-emitter (Scheme 1B). Compared with complex surface post-modifications, this strategy utilizing the intrinsically features of porous MOFs are expected to be much simpler and more efficient. Finally, barium-based MOFs with good stability should have high binding affinity towards ^224^Ra^2+^, due to the chemical similarity between alkaline earth metals. Since the radiopharmaceutical is always at trace amount, the biosafety of the barium-based MOFs can also be guaranteed. With all these in minds, we applied systematically computational investigations and radiochemical experiments, including density functional theory (DFT), X-ray photoelectron spectroscopy (XPS) and X-ray absorption spectroscopy (XAS), to determine the radioactivity localization ability of the radionuclide trap consisting of the side-chain phenol and carboxylic groups of the nanoscale AEMOF-6 towards the key decay daughters of ^224^Ra. Then the radioactivity biodistribution, antitumor effect and endoradiotherapy safety of the obtained ^224^Ra-AEMOF-6@CS were systematically investigated. We have demonstrated that the excellent radionuclide capturing ability enables the ^224^Ra-labeled radiopharmaceutical to have ideal *in vivo* radioactivity localization ability, good antitumor effect and high biosafety.

## Results and Discussion

### DFT calculations on the coordination of Ra by the metal nodes of AEMOF-6

**Figure 1:**
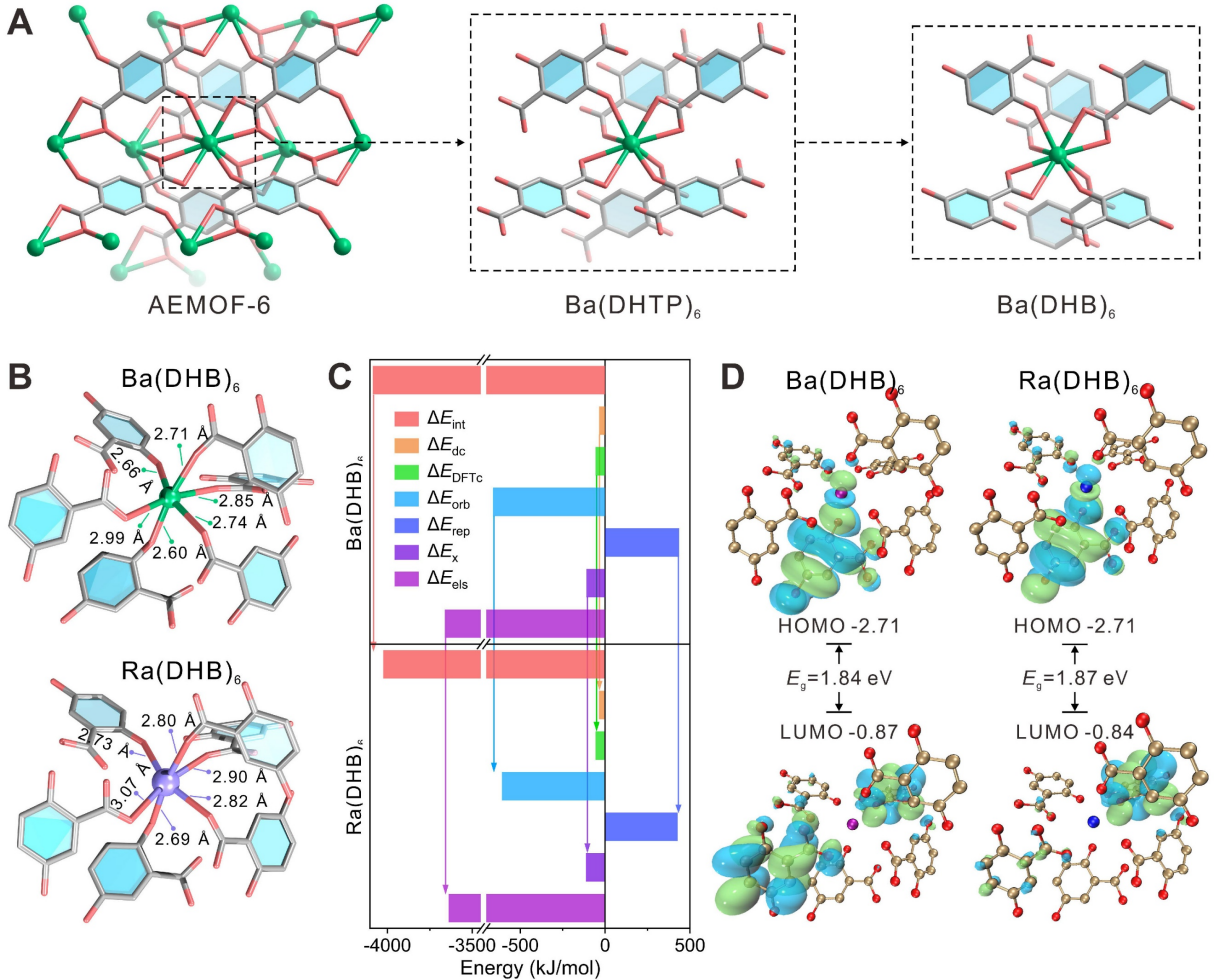
(A) Structures of AEMOF-6, Ba(DHTP)_6_, and Ba(DHB)_6_ complexes. (B) Theoretically optimized structures of Ba(DHB)_6_ and Ra(DHB)_6_ complexes. (C) EDA of Ba(DHB)_6_ and Ra(DHB)_6_ complexes, including interaction energy (Δ*E*_int_), dispersion correction energy (Δ*E*_dc_), DFT correlation energy (Δ*E*_DFTc_), orbital interaction energy (Δ*E*_orb_), Pauli repulsion energy (Δ*E*_rep_), exchange energy (Δ*E*_x_), and electrostatic energy (Δ*E*_els_). (D) Diagrams of the LUMO and HOMO for Ba(DHB)_6_ and Ra(DHB)_6_ complexes with the corresponding Δ*E*_LUMO-HOMO_ gaps (The isosurface values are set to 0.01 au). The influence of water molecules and counterions had been omitted to emphasize the coordination interactions between the examined cations and (DHB)_6_.

The DFT calculation 41-43 results confirm that AEMOF-6 is a suitable nanocarrier for ^224^Ra labeling. The structure of AEMOF-6 was simplified as Ba(DHB)_6_ for computational investigations (Figure 1A). As shown in Figure 1B, the optimized structures of Ra(DHB)_6_ and Ba(DHB)_6_ exhibited remarkable similarity. The Δ*E* values for Ra(DHB)_6_ and Ba(DHB)_6_ were determined to be -5792.52 and -5730.65 kJ/mol, respectively, with a slight difference of 1.15%, indicating nearly identical coordination affinity of AEMOF-6 towards Ra²⁺ and Ba²⁺ (Tables S1 and S2). Furthermore, the Ra-O bond lengths in Ra(DHB)_6_ were measured as 2.69 ~ 3.07 Å (2.84 ± 0.14 Å), while those in Ba(DHB)_6_ were recorded as 2.60 ~ 3.99 Å (2.76 ± 0.14 Å). It was observed that the average bond length of Ra-O was slightly longer than Ba-O by 0.077 Å, which could be attributed to their differences in ionic radius (Ra^2+^: 1.48 Å; Ba^2+^: 1.42 Å 44). Energy decomposition analysis (EDA) 45-46 (Figure 1C) further reveals highly similar interactions between AEMOF-6 and Ra/Ba. The Δ*E*_int_, Δ*E*_dc_, Δ*E*_DFTc_, Δ*E*_orb_, Δ*E*_rep_, Δ*E*_x_, and Δ*E*_els_ values of Ra(DHB)_6_ corresponded to 98.49%, 104.75%, 100.67%, 92.03%, 97.93%, 102.36%, and 99.39% of those for Ba(DHB)_6_, respectively. The interaction energies between Ra(DHB)_6_ and Ba(DHB)_6_ were determined to be comparable (<5% variation), except for Δ*E*_orb_ showing a 7.93% difference. Given that Δ*E*_orb_ originates from energy variations induced by the mixture of the occupied and unoccupied orbitals between different fragments, primarily reflecting covalent interactions 45, these results indicate that the interaction differences between Ra and Ba are predominantly governed by coordination covalent effects. Molecular orbital analysis 47 results (Figure 1D) demonstrate that Ra(DHB)_6_ possesses same HOMO energy but slightly lower LUMO energy compared to Ba(DHB)_6_, indicating enhanced electrophilicity. Crucially, the Δ*E*_LUMO-HOMO_ energy gaps were calculated as 1.87 eV for Ba(DHB)_6_ and 1.84 eV for Ra(DHB)_6_, differing by only 0.03 eV. According to Koopmans' approximation theory 48, molecular softness is inversely proportional to the Δ*E*_LUMO-HOMO_ gap. The smaller energy gap observed in Ra(DHB)_6_ suggests greater molecular softness and higher chemical reactivity. Comprehensive analysis of binding energies, bond lengths, energy decomposition, and Δ*E*_LUMO-HOMO_ gaps confirms the high structure similarity between Ra(DHB)_6_ and Ba(DHB)_6_ complexes. However, intrinsic differences in metallic properties between Ra and Ba may result in slightly reduced stability for Ra(DHB)_6_ compared to Ba(DHB)_6_.

### Coordination effect of AEMOF-6 towards the key decay daughters of ^224^Ra

**Figure 2:**
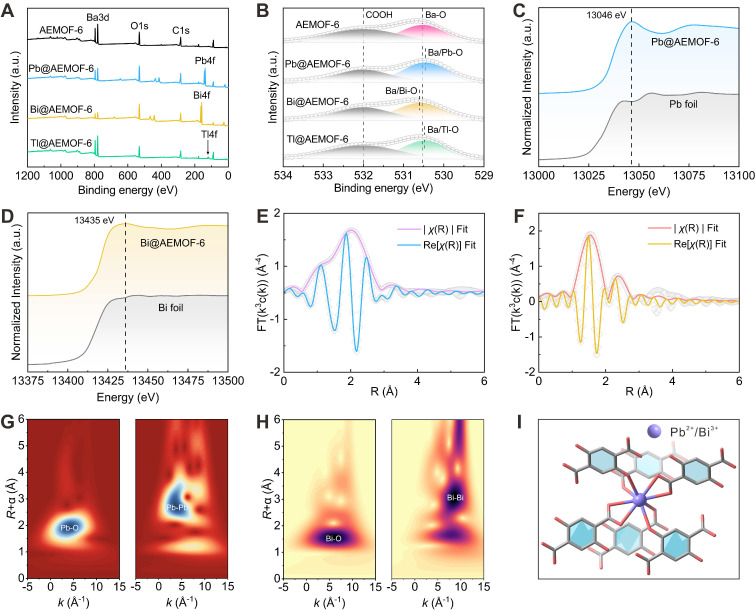
XPS (A) survey spectra and (B) O1s high-resolution spectra of AEMOF-6, Pb@AEMOF-6, Bi@AEMOF-6, and Tl@AEMOF-6. The background-subtracted and normalized L3-edge XANES spectra obtained from (C) Pb@AEMOF-6 and (D) Bi@AEMOF-6. FT of (E) Pb@AEMOF-6 (k-range: 3.5~8 Å^-1^ and R-range: 1~3.5 Å) and (F) Bi@AEMOF-6 (k-range: 3~9 Å^-1^ and R-range: 1~3.5 Å) EXAFS spectra with the best-fit EXAFS models. WT-EXAFS of (G) Pb@AEMOF-6 and Pb foil, (H) Bi@AEMOF-6 and Bi foil. (I) Structures proposed for the DHTP and Pb^2+^/Bi^3+^ in which Pb^2+^/Bi^3+^ is 8-coordinated with O from the carboxylate and side-chain phenol groups.

We then verified the synergetic coordination effect of the radionuclide trap consisting of the side-chain phenol and carboxylic groups of AEMOF-6 towards the key decay daughters of ^224^Ra. In the decay chain of ^224^Ra, ^212^Pb (*T*_1/2_=10.6 h), ^212^Bi (*T*_1/2_=60.6 m), and ^208^Tl (*T*_1/2_=3.0 m) have significantly longer half-lives than ^220^Rn (*T*_1/2_=55.6 s), ^216^Po (*T*_1/2_=0.2 s), and ^212^Po (*T*_1/2_=0.3 μs), thereby posing a higher risk of migration from target sites to healthy tissues 11. Specifically, ^212^Pb and ^212^Bi emit high-energy α particles either directly or through the decay of their daughter radionuclides. Although ^208^Tl is a β radionuclide that decays directly into a stable radionuclide, its decay is accompanied by an intense high-energy γ ray (2.6 MeV, 99%). Moreover, Pb, Bi, and Tl commonly appear as long-lived daughter radionuclides in the decay chains of other common α-radionuclides, such as ^223^Ra and ^225^Ac (Figure S1). Therefore, evaluating the capability of AEMOF-6 to capture Pb, Bi, and Tl is essential for achieving effective *in vivo* radioactivity confinement of typical α radionuclides. Firstly, the coordination interactions between AEMOF-6 and Tl, Bi, and Pb were confirmed through XPS analysis of the chemical composition before and after adsorption. In the survey spectra of XPS for Pb@AEMOF-6, Bi@AEMOF-6, and Tl@AEMOF-6 (Figure 2A), characteristic peaks corresponding to Bi4f, Pb4f, and Tl4f were observed at 138.08, 159.08, and 118.08 eV, respectively, confirming the successful adsorption of Pb, Bi, and Tl by AEMOF-6. Meanwhile, as shown in the high-resolution spectra of Pb4f, Bi4f, and Tl4f (Figure S3), the oxidation states of Pb, Bi, and Tl are +2, +3, and +1 in Pb@AEMOF-6, Bi@AEMOF-6, and Tl@AEMOF-6, respectively. Furthermore, in the O1s spectra of Pb@AEMOF-6, Bi@AEMOF-6, and Tl@AEMOF-6 (Figure 2B), the binding energy of the Ba-O characteristic peak shifted from 530.53 to 530.46, 530.60, and 530.48 eV, respectively. These shifts indicate that Pb^2+^, Bi^3+^, and Tl^+^ were adsorbed by AEMOF-6 through coordination with O atoms from -COOH and -OH groups.

Besides, XAS analysis also confirmed the coordination interactions of AEMOF-6 with Pb and Bi. As depicted in Figures 2C-D, a single peak associated with related metal centers was observed in the front edges of both Pb@AEMOF-6 (13047 eV) and Bi@AEMOF-6 (13436 eV) complexes, indicating that either Pb^2+^ or Bi^3+^ has only one oxidation state in corresponding compounds 49-52. Corresponding Fourier transform (FT) data (Figures 2E-F) exhibited a single peak at 2.02 Å for Pb-O and 1.53 Å for Bi-O, with both signals being predominantly ascribed to O scatterers within the first coordination shell. Artemis software analysis revealed 8-coordinate geometries for both metal ions (Figure S4, Table S3), with average bond distances of 1.79~2.13 Å for Pb-O and 2.24~2.53 Å for Bi-O. Wavelet transform (WT) analysis (Figure 2G-H) confirmed distinct Pb-O 53-54 and Bi-O 55 bonding signals, fully consistent with EXAFS results. These results suggest that the coordination localization effect of AEMOF-6 is mechanistically realized through 8-oxygen coordination of the -COOH and -OH groups of AEMOF-6 to these cations (Figure 2I). It can be expected that the permanent porosity of AEMOF-6 can align the carboxylate and side-chain phenol groups as a coordination network capable of recapturing the detached recoiling decay daughters.

### Preparation and characterization of ^224^Ra-AEMOF-6@CS

**Figure 3:**
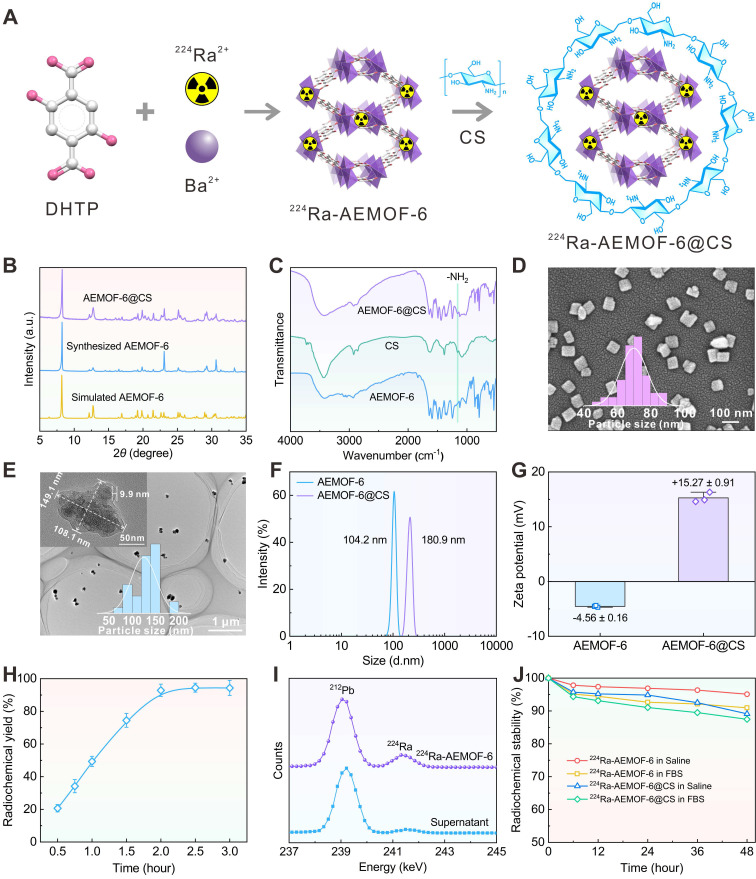
(A) Schematic illustration of the preparation of ^224^Ra-AEMOF-6@CS. (B) PXRD patterns of AEMOF-6 and AEMOF-6@CS. (C) FTIR spectra of AEMOF-6, CS, and AEMOF-6@CS. (D) SEM image of AEMOF-6. (E) TEM image of AEMOF-6@CS. (F) DLS diameter and (G) Zeta potentials of AEMOF-6 and AEMOF-6@CS. (H) Radiochemical yield as a function of reaction time and (I) γ spectra of the precipitate and supernatant after 2 h of reaction of ^224^Ra-AEMOF-6. (J) Radiochemical stability of ^224^Ra-AEMOF-6 and ^224^Ra-AEMOF-6@CS after incubation in saline and 20% FBS.

^224^Ra doping and chitosan (CS) modification can yield ^224^Ra-AEMOF-6@CS with high radiolabeling conversion and good biocompatibility (Figure 3A). Before radiochemistry, the nanoscale AEMOF-6 was prepared and characterized systematically. The experimental PXRD pattern of the synthesized AEMOF-6 is consistent with the simulated one 36, and exhibits characteristic peaks at 8.2° (110), 12.7° (400), 23.1° (130), and 30.6° (040), confirming the successful synthesis of AEMOF-6 (Figure 3B). After the modification of CS, no significant differences were observed between the PXRD patterns of AEMOF-6@CS and AEMOF-6, indicating that the crystalline structure of AEMOF-6 remained intact. Meanwhile, FTIR analysis (Figure 3C) reveals a new absorption peak around 1157 cm^-1^ in AEMOF-6@CS, attributed to the asymmetric stretching vibration of glycosidic bonds in CS, further verifying the successful synthesis of AEMOF-6@CS. SEM analysis demonstrates that the synthesized AEMOF-6 particles exhibit a block-like morphology with dimensions of approximately 63.2 × 68.7 nm (Figure 3D). More importantly, AEMOF-6 could maintain its original geometric micromorphology after loading with Pb, Bi, and Tl (Figure S5), indicating that it should have good structure stability during the adsorption of corresponding daughters. CS modification increased the particle size of AEMOF-6@CS to 108.1 × 149.1 nm, with a CS layer thickness of ~9.9 nm (Figure 3E). The hydrodynamic diameters of AEMOF-6 and AEMOF-6@CS were determined to be 104.2 and 180.9 nm by DLS analysis, respectively (Figure 3F). Meanwhile, compared to AEMOF-6, the zeta potential of AEMOF-6@CS significantly increased from -4.56 ± 0.19 to 15.27 ± 0.91 mV (Figure 3G). The negative surface charge of the pristine AEMOF-6 supposedly originated from the deprotonated -Ba-O clusters or residual -COOH groups of the ligand. The significant increase in zeta potential after CS modification was assigned to the protonation of amino groups on CS molecular chains under neutral or weakly acidic conditions 56.

AEMOF-6 has shown excellent ability to encapsulate ^224^Ra^2+^ and its decay daughters. Regarding ^224^Ra radiolabelling, the radiochemical yield of ^224^Ra-AEMOF-6 can achieve 92.87 ± 3.75% within 2 h and tends to keep constant with prolonging the reaction time (Figure 3H). In the γ spectrum of the product (Figure 3I), characteristic γ peaks of ^224^Ra (240.99 keV) and its daughter nuclide ^212^Pb (238.63 keV) were detected. The significantly weaker γ intensity of ^224^Ra in the spectrum of supernatant confirmed the efficient labeling of ^224^Ra by AEMOF-6. The radiochemical purity of ^224^Ra-AEMOF-6, as tested by Radio thin-layer chromatography (Radio-TLC), was calculated to be 94.75% (Figure S6). After 48 h of incubation in saline and 20% fatal bovine serum (FBS) in phosphate-buffered saline (20% FBS), the radiochemical stability of ^224^Ra-AEMOF-6 remained as high as 95.10% and 90.96%, respectively (Figure 3J). In addition, some previously reported nanocarriers, such as TiO_2_ nanoparticles 20, exhibit obvious leak (20~40%) of the daughter radionuclides from ^223^Ra in the testing medium within 48 h. In comparison, ^224^Ra-AEMOF-6 in this work demonstrate superior radioactivity encapsulation. This indicates that the proposed strategy exploiting radionuclide trap preconstructed in nanoscale AEMOF-6 is more effective for localizing daughter radionuclides relative to conventional physical encapsulations. For any radioactivity encapsulation method, further verifications are still necessary before they step into clinical practice. The lower stability of ^224^Ra-AEMOF-6 in 20% FBS was caused by the high abundance of albumin and globulins in FBS, which may partially capture ^224^Ra and its daughter nuclides, leading to reduced radiochemical stability. The modification of CS slightly reduced the radiochemical stability of ^224^Ra-AEMOF-6@CS to some extent, but still shows good radioactivity encapsulation effect (>87%) over the observation period.

^224^Ra-AEMOF-6@CS exhibits significantly enhanced cancer cell binding and improved the internalization ability toward 4T1 cell line (Figures S7). This may be assigned to the biocompatible and protonated surface of ^224^Ra-AEMOF-6@CS, which is favorable for cancer cell to endocytose corresponding nanoparticles. Benefiting from the high cellular binding and internalization efficiency of ^224^Ra-AEMOF-6@CS, a significantly enhanced inhibitory effect on cell viability was observed in cytotoxicity assays (Figure S8). In contrast, AEMOF-6@CS carrier itself produced no significant cytotoxicity at a wide concentration range of 5~60 μg/mL, confirming that the observed cell death was specifically caused by high linear energy transfer (LET) radiation from the decay of ^224^Ra. These results highlight the therapeutic potential of ^224^Ra-AEMOF-6@CS as an alpha-particle delivery system.

### *In vivo* radioactivity localization effect of ^224^Ra-AEMOF-6@CS

**Figure 4:**
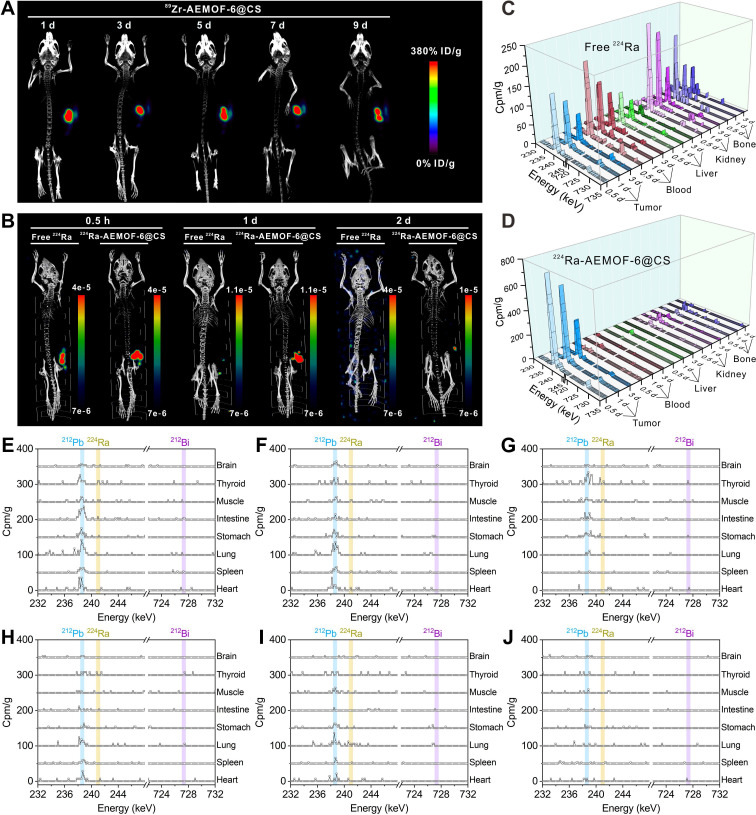
(A) Micro-PET/CT imaging in subcutaneous 4T1 tumor-bearing mice at different time points after *i.t.* injection of ^89^Zr-AEMOF-6@CS. (B) Micro-SPECT/CT imaging in subcutaneous 4T1 tumor-bearing mice at different time points after *i.t.* injection of free ^224^Ra or ^224^Ra-AEMOF-6@CS. The γ-energy spectrum diagram of tumor, blood, liver, kidney, and bone of subcutaneous 4T1 tumor-bearing mice after *i.t.* injection of (C) free ^224^Ra and (D) ^224^Ra-AEMOF-6@CS at 0.5, 1, and 3 d *p.i.* The γ-energy spectrum diagram of heart, spleen, lung, stomach, intestine, muscle, thoroid and brain of subcutaneous 4T1 tumor-bearing mice after *i.t.* injection of free ^224^Ra at (E) 0.5, (F) 1, and (G) 3 d *p.i.* The γ-energy spectrum diagram of heart, spleen, lung, stomach, intestine, muscle, thoroid and brain of subcutaneous 4T1 tumor-bearing mice after *i.t.* injection of free ^224^Ra-AEMOF-6@CS at (H) 0.5, (I) 1, and (J) 3 d *p.i.*

Prior to investigating the *in vivo* radioactivity localization effect of ^224^Ra-AEMOF-6@CS, AEMOF-6@CS was labeled with the positron-emitting nuclide ^89^Zr (*T*_1/2_ = 3.3 d), and micro-PET/CT imaging was performed to assess the *in vivo* stability. As shown in Figure 4A, ^89^Zr-AEMOF-6@CS remained localized within the tumor at 1, 3, 5, 7, and 9 d post-injection (*p.i.*), with no detectable diffusion to normal tissues or organs, demonstrating excellent *in vivo* stability of AEMOF-6@CS. Besides, ^224^Ra and its decay daughter ^212^Pb emit γ-rays at 240.99 keV (abundance: 4.10%) and 238.63 keV (abundance: 43.6%), respectively, which align well with the ideal energy window (~200 keV) for micro-SPECT/CT imaging. However, as an α-emitter, ^224^Ra requires strict dose control to mitigate radiation hazards, raising questions about its imaging capability at conventional therapeutic doses. To address this, micro-SPECT/CT imaging of mice receiving intratumoral injections of 37.0 kBq free ^224^Ra and ^224^Ra-AEMOF-6@CS was conducted to evaluate the imaging potential of ^224^Ra-AEMOF-6@CS and the radioactivity localization efficacy (Figures 4B and S9). Comparatively, the SPECT signal of the group receiving free ^224^Ra decreased significantly at 1 d *p.i.*, while the group receiving ^224^Ra-AEMOF-6@CS maintained clear tumor-specific signals even at 2 d *p.i*. These results not only suggest that ^224^Ra-AEMOF-6@CS administered at 37.0 kBq exhibits promising SPECT/CT imaging potential, but also indicate the superior *in vivo* radioactivity localization ability of ^224^Ra-AEMOF-6@CS.

*In vivo* biodistribution studies confirmed the enhanced radioactivity localization efficacy of ^224^Ra-AEMOF-6@CS. As shown in Figure 4C, mice after *i.t.* injection of free ^224^Ra resulted in detectable γ-peaks for ^224^Ra (240.99 keV) and its decay daughter ^212^Pb (238.63 keV) and ^212^Bi (727.33 keV) within the tumor at 0.5, 1, and 3 d, with uptake values near 239 keV measured as 147.9, 118.3, and 73.9 cpm/g, respectively. Meanwhile, γ spectra of blood, liver, kidney, and bone revealed significant leakage of daughter radionuclides into normal organs/tissues (Figure S10). In contrast, γ spectra of the ^224^Ra-AEMOF-6@CS group (Figure 4D) showed predominant localization of ^224^Ra, ^212^Pb, and ^212^Bi within the tumor, with uptake values near 239 keV reaching 741.7, 617.3, and 310.5 cpm/g at 0.5, 1, and 3 d *p.i.*, representing 5.0, 5.2, and 4.2 times higher than those observed in the group receiving free ^224^Ra, respectively. Notably, there were no significantly ^212^Pb and ^212^Bi peaks detected in blood, liver, kidney, lung, and bone of the group receiving ^224^Ra-AEMOF-6@CS (Figures 4E-J and S11), indicating the improved *in vivo* radioactivity localization ability.

### Therapeutic efficacy of ^224^Ra-AEMOF-6@CS

**Figure 5:**
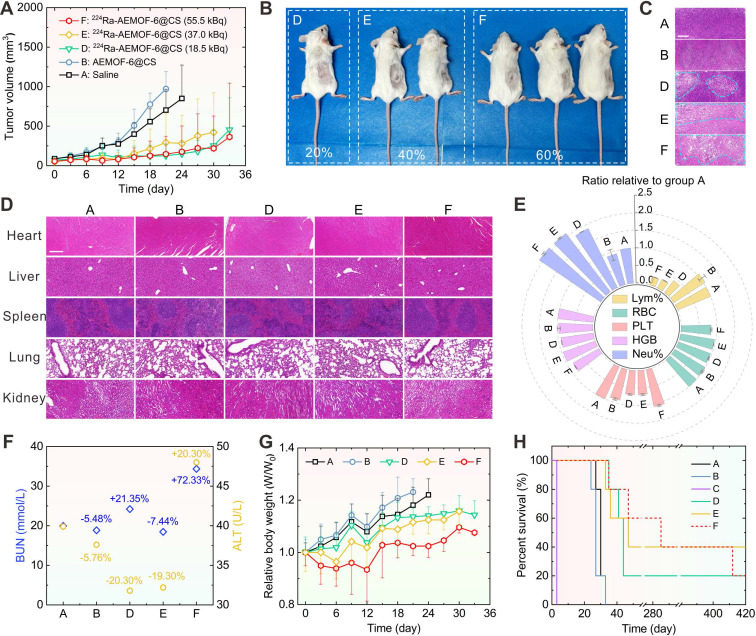
(A) Tumor volume change, (B) the optical photos of group D, E, F at 36 d *p.i.*, (C) H&E staining of tumor issues (Scale bar: 100 μm), the added blue dashed lines are used to highlight the tumor tissues with significant morphological changes, (D) H&E staining images of heart, liver, spleen, lung, and kidney (Scale bar: 100 μm), (E) Blood routine examination for Lymphocyte Percentage (Lym%), Red Blood Cell Count (RBC), Platelet Count (PLT), Hemoglobin (HGB), and Neutrophil Percentage (Neu%), (F) Hepatic (Blood urea nitrogen, BUN) and Renal (Alanine aminotransferase, ALT) function levels at 15 d *p.i.*, (G) relative body weight change, (H) Kaplan-Meier survival plot of the six groups [(A) Saline, (B) AEMOF-6@CS, (C) 37.0 kBq free ^224^Ra, (D) 18.5 kBq ^224^Ra-AEMOF-6@CS, (E) 37.0 kBq ^224^Ra-AEMOF-6@CS, and (F) 55.5 kBq ^224^Ra-AEMOF-6@CS] during the therapeutic period (*n* = 5). Samples for H&E staining and immunofluorescence staining from the five groups were collected at 15 d *p.i*. The blood samples were collected at day 15 after treatment.

The therapeutic efficacy of ^224^Ra-AEMOF-6@CS was evaluated *in vivo*. Notably, mice receiving intratumorally (*i.t.*) administration of 37.0 kBq free ^224^Ra developed severe skin ulceration at 3 d *p.i.* (Figure S14), necessitating immediate euthanasia. As depicted in Figures 5A and S12, tumor growth in group D (18.5 kBq), E (37.0 kBq), and F (55.0 kBq) where mice treated with different doses of ^224^Ra-AEMOF-6@CS was significantly slower than those receiving normal saline (group A) and cold AEMOF-6@CS (group B). The tumor inhibition rates of group D, E, and F reached 68.7%, 61.7%, and 73.5% at 15 d *p.i.*, respectively. At 36 d *p.i.*, 20%, 40%, and 60% of mice in group D, E, and F achieved complete tumor eradication without recurrence over 400 days, respectively (Figure 5B). Furthermore, dose-dependent morphological alterations and necrosis were observed in H&E staining of tumor tissues (Figure 5C), with groups D, E, and F exhibiting a progressive increase in cellular shrinkage and necrotic severity.

In addition to the demonstrated dose-dependent therapeutic superiority, ^224^Ra-AEMOF-6@CS also exhibited favorable biosafety. As expected, no significant damage was observed in liver, spleen, kidney, heart, or lung tissues of groups D, E, and F through H&E staining analysis (Figure 5D). However, hematological analysis revealed potential radiation-induced inflammatory responses during the treatment period. Blood routine examination results showed that the neutrophil percentages in groups D, E, and F were 1.87 ± 0.01, 2.08 ± 0.02, and 2.10 ± 0.03 folds higher than that in group A, respectively, indicating a dose-dependent increase in radiation-induced inflammation (Figure 5E). Meanwhile, lymphocyte percentages decreased proportionally with rising neutrophil levels. Notably, group F also showed slight reductions in red blood cell count (0.84 ± 0.02 relative to group A) and hemoglobin concentration (0.83 ± 0.01 relative to group A), but remained within normal ranges. Function analysis of liver and kidney reveals BUN levels of 19.9, 18.8, 24.2, 18.4, and 34.36 mmol/L, and ALT levels of 39.9, 37.6, 31.8, 32.2, and 46.0 U/L for Groups A, B, D, E, and F, respectively (Figure 5F). Significantly elevated BUN and ALT levels were observed in group F compared to group A, indicative of potential inflammatory responses or cellular injury. Given that the AEMOF-6@CS carrier demonstrated excellent biosafety *in vitro* and *in vivo*, showing no significant cytotoxicity, tissue damage, or abnormal blood parameters, the inflammatory reactions in the ^224^Ra-AEMOF-6@CS groups were thus attributable to radiation effects of ^224^Ra rather than the AEMOF-6@CS carrier itself. Although group E showed minor decreases in BUN and ALT levels, these fluctuations may reflect some physiological variability without pathological significance. Relative weights of 1.18 ± 0.07, 1.23 ± 0.05, 1.14 ± 0.03, 1.12 ± 0.05, and 1.02 ± 0.04 for group A, B, D, E, and F at 21 d *p.i.* were measured, respectively, indicating slower weight gain in groups receiving ^224^Ra-AEMOF-6@CS with dose-dependent suppression (Figures 5G and S13). Despite partial weight recovery in the group F at 12 d *p.i.*, these findings emphasize the necessity for further optimizing administered dose and fractioned injection to minimize potential toxicity in clinical applications. Driven by its excellent tumor suppressive efficacy and satisfactory biosafety, the median survival durations for groups D, E, and F were extended to 44, 47, and 285 d, respectively (Figure 5H), corresponding to 1.6, 1.7, and 10.6 times that of group A (27 d). Overall, these results demonstrate a dose-dependent antitumor response for ^224^Ra-AEMOF-6@CS within the administered dose range of 18.5~55.5 kBq, where both tumor inhibition and endoradiotherapy biosafety can be balanced.

In summary, the strategy proposed in this work that exploits a radionuclide trap preconstructed in nanoscale AEMOF-6 represents a highly promising approach for achieving efficient radiolabeling and *in vivo* radioactivity localization in ^224^Ra-based endoradiotherapy. Although biological barriers pose significant challenges to the effective delivery of nanomedicines and hinder their successful accumulation at disease sites, localized administration methods can selectively enhance the accumulation and penetration of nanodrugs within tumors. This holds great therapeutic potential for specific cancer types and primary tumors not suitable for surgical resection. Local administration not only protects healthy tissues and reduces adverse effects but also enhances antitumor efficacy by increasing intratumoral nanodrug concentration and prolonging retention time 57. For example, Mengdie Yang et al. 58 have found that ^212^Pb-labeled hydrogel nanoparticles (^212^Pb-HNPs) could induce oxidative stress in tumor tissues, which shows considerable potential for tumor treatment. Sara Westrøm et al 59 utilized CaCO_3_ particles to encapsulate ^224^Ra and found that intraperitoneal injection of corresponding nanoradiopharmaceutical could suppress peritoneal and ovarian cancers. Furthermore, nanocarriers administered intravenously are more like to accumulate in liver, lungs, and spleen which are rich in reticuloendothelial system (RES). Leveraging this characteristic to use nanodrugs to treat cancers which have metastasized to RES-rich organs or tissues presents another feasible and innovative therapeutic pathway. However, when administered intravenously, particular attention must be paid to further optimizing the particle size, *in vivo* stability, and clearance efficiency of nanodrugs to ensure the accumulation of α-radionuclides at the tumor site.

## Conclusions

We proposed a strategy exploiting radionuclide trap preconstructed in nanoscale AEMOF-6 to achieve efficient radiolabeling and* in vivo* radioactivity localization of ^224^Ra endoradiotherapy. A ^224^Ra-labeled radiopharmaceutical was successfully synthesized, demonstrating high radiochemical yield, radiochemical purity, and *in vitro* stability. Integrated micro-PET/CT and micro-SPECT/CT imaging, complemented by biodistribution analyses, validated the robust stability and radioactivity localization capability of the AEMOF-6@CS nanocarrier *in vivo*. The developed ^224^Ra-AEMOF-6@CS exhibited excellent antitumor efficacy and favorable biosafety profiles during cancer endoradiotherapy in a mouse model. Future investigations will focus on optimizing therapeutic protocols, including administered dose, fractioned administration, and long-term toxicity.

## Materials and Methods

### DFT calculations

Geometry optimizations were explored by density functional theory (DFT) calculations, which were performed using the Perdew-Burke-Ernzerhof (PBE) functional implemented in Gaussian 09. C, H, and O atoms were treated by the 6-31G(d) basis set 60. Metal atoms were modeled using small-core quasi-relativistic pseudopotentials for the related atoms (Ba: ECP46MWB; Ra: ECP78MDF) and the associated basis sets. All species were optimized in aqueous solution using the polarizable continuum model (PCM) 61. The more precise electron energy was calculated with the PBEPBE/6-311+G(d,p) basis set. Frequency analysis was performed on all optimized stationary points to identify their nature as a minimum and the Gibbs free energy was provided. To further analyze the interactions in Ba(DHB)_6_ and Ra(DHB)_6_, energy decomposition analysis was performed based on the sobEDA method 45 using the PBE functional and a mixed basis set (C, H, O: def2-TZVP basis set; Ba: ECP46MWB; Ra: ECP78MDF). The relevant calculations were carried out using the Multiwfn software 46. Furthermore, the optimized structures have been used to calculate the highest occupied molecular orbital (HOMO) and lowest unoccupied molecular orbital (LUMO) energy by Gaussian 09W software. The HOMO-LUMO orbital diagram was drawn using Multiwfn 46 and VMD 47 software.

### Materials

Warning! ^224^Ra and ^89^Zr and these decay daughters present strong biotoxicity and require special radioprotective precautions. The research was carried out under radiation safety conditions permitting.

2,5-Dihydroxyterephthalic acid (DHTA), Ba(NO_3_)_2_, and N,N-Dimethylacetamide (DMAc) were obtained from Sigma-Aldrich. Chitosan (CS, M_w_=30000) was purchased from Shanghai Macklin Biochemical Co., Ltd. FBS and RPMI 1640 were purchased from Tianhang Biotechnology Co., Ltd. (Hangzhou, China). The ^224^Ra solution used in this study was eluted from a ^228^Th/^224^Ra generator 2, 16. Its radionuclidic purity was determined to be greater than 99.50% (Figure S2). ^89^Zr was produced through CS-30 cyclotron according to the published protocol 62.

### Synthesis

Synthesis of AEMOF-6@CS. First, AEMOF-6 nanoparticles were synthesized according to the method reported in reference 36: Ba(NO_3_)_2_ (28.74 mg, 0.11 mmol) was weighed and added to deionized water (2 mL), followed by ultrasonication for 30 min to ensure complete dissolution. Subsequently, DHTA (35.66 mg, 0.18 mmol) was weighed and dissolved in DMAc (7 mL) with 5 min of ultrasonication. The two solutions were then mixed, sealed, and reacted in a 60 °C ultrasonic reactor for 90 min. After completion, the product was isolated by centrifugation, washed, and dried to obtain AEMOF-6 powder. Next, the synthesized AEMOF-6 powder (2.00 mg) was mixed with CS (1.00 mg) in a mixed solvent of ethanol/1% acetic acid solution (volume ratio 4:1). The reaction was allowed to proceed under magnetic stirring at room temperature for 60 min. Finally, the product was washed with ethanol and deionized water to yield AEMOF-6@CS.

Synthesis of ^224^Ra-AEMOF-6@CS. The synthesis procedure for ^224^Ra-AEMOF-6 was similar to that of AEMOF-6, with the only modification being the addition of ^224^Ra(NO_3_)_2_ solution during the AEMOF-6 synthesis. After the reaction, ^224^Ra-AEMOF-6 was obtained *via* centrifugation, and its radiochemical yield was determined by measuring the γ spectra of the precipitate (^224^Ra-AEMOF-6) and the supernatant (free ^224^Ra) using an HPGe detector. The yield was calculated based on the 240.99 keV γ emission peak of ^224^Ra. Subsequently, following the method described in Section 1.4.1, the synthesized ^224^Ra-AEMOF-6 was mixed with CS in an ethanol/1% acetic acid solution. Upon completion of the reaction, ^224^Ra-AEMOF-6@CS was obtained and washed with ethanol and deionized water.

Synthesis of ^89^Zr-AEMOF-6@CS. The synthesis method for ^89^Zr-AEMOF-6@CS was analogous to that of ^224^Ra-AEMOF-6@CS, except that the ^224^Ra(NO_3_)_2_ solution was replaced with ^89^ZrCl_4_ solution (produced using the CS-30 cyclotron at Sichuan University). After the reaction, ^89^Zr-AEMOF-6@CS was obtained.

### Tumor model

Balb/c mice (female, 5 weeks) were purchased from Chengdu Dossy Experimental Animals Co., LTD. (Chengdu, China). 4T1 cells (2 × 10^6^) suspended in 150 μL of PBS were subcutaneously injected into the right back of each Balb/c mouse. All animal studies were conducted in accordance with the institutional ethics committee regulations and guidelines on animal welfare and approved by the Animal Welfare and Ethics Committee of Sichuan University.

### Micro-PET/CT imaging

For micro-PET/CT imaging, each mouse intratumorally injected with 2.78 MBq of ^89^Zr-AEMOF-6@CS was anesthetized with 2% isoflurane for making whole-body micro-PET/CT images of the at 1, 3, 5, 7, and 9 d *p.i*. using an Inveon micro-PET/CT scanner (Preclinical Solutions; Siemens Healthcare Molecular Imaging, Knoxville, TN, USA). Each scan was completed within 15 min. The obtained images were reconstructed using three-dimensional ordered-subset expectation maximization (3D OSEM) and then processed using Osirix MD.

### Micro-SPECT/CT imaging

To evaluate the biodistribution of free ^224^Ra and ^224^Ra-AEMOF-6@CS in tumor and normal tissues, 4T1 tumor-bearing mice were intratumorally injected with either 37.0 kBq of free ^224^Ra or 37.0 kBq of ^224^Ra-AEMOF-6@CS in saline solution. Micro-SPECT/CT imaging was conducted at 0.5 h, 1 d, and 2 d *p.i*., under anesthesia with 2% isoflurane utilizing an ultrahigh-resolution micro-SPECT system (Netherlands) equipped with a low-energy all-purpose collimator. The energy window was configured to 240 keV. Data acquisition proceeded at a scanning speed of 10 min per frame, with each subject undergoing a total of two frames. All images underwent reconstruction using the ordered-subset expectation maximization (OSEM) algorithm, incorporating scatter and attenuation corrections, through the MIlabs reconstruction software (Utrecht, Netherlands). Subsequently, the images were reconstructed using the software for final visualization purposes.

### Biodistribution

Typically, when tumor volume reached ~200 mm^3^, mice bearing 4T1 tumors were intratumorally injected with 14.8 kBq free ^224^Ra or ^224^Ra-AEMOF-6@CS. At 0.5, 1, and 3 d post-injection (*n* = 3), the mice were euthanized by isoflurane. In the decay chain of ^224^Ra, ^212^Pb (*T*_1/2_ = 10.6 h) and ^212^Bi (*T*_1/2_ = 60.55 min) emit γ rays with energies of 238.63 keV (43.6%) and 727.33 keV (6.67%), respectively, which can be detected by HPGe detector. Given the short half-lives of ^212^Pb and ^212^Bi, the measuring time for each sample must be strictly controlled. Specifically, each dissected organ or tissue was immediately placed in a clean polypropylene centrifuge tube for weighing, of which the radioactivity (counts/min/g (cpm/g)) was then instantly analyzed using an HPGe detector, with a measuring time of 60 s per sample. In addition, an FH463B γ well-type scintillation intelligent detector was used to measure the total radioactivity counts of each sample (10 s per sample), and the decay-correction radioactivity uptake (% ID/g) was calculated accordingly. Note: all samples were placed for 6 h to ensure that decay equilibrium between ^224^Ra, ^212^Pb, and ^212^Bi was established before measuring.

### *In vivo* therapeutic effect

To evaluate the therapeutic effect of ^224^Ra-AEMOF-6@CS, 4T1 tumor-bearing mice were randomly assigned to five groups (7 mice per group) to receive *i.t.* injection of: (A) saline, (B) AEMOF-6@CS (5 mg/Kg), (C) 37.0 kBq free ^224^Ra, (D) 18.5 kBq ^224^Ra-AEMOF-6@CS, (E) 37.0 kBq ^224^Ra-AEMOF-6@CS and (E) 55.5 kBq ^224^Ra-AEMOF-6@CS. The treatment was conducted when tumor volume reached 40~60 mm^3^. The tumor volume was measured blindly with an electronic caliper every 3 days until the subjects reached the endpoint (tumor volume >1000 mm^3^, death, ulcerating tumor tissue, or >20% weight loss). The calculation formula was as follows: tumor volume (mm^3^) = (length × width^2^)/2. The body weight of the corresponding subjects was recorded to evaluate the potential whole-body toxicity during the treatment. Two mice from each group were sacrificed at 15 d post injection, of which the blood (~0.8 mL) was drawn for routine examination.

## Supplementary Material

Supplementary methods, figures and tables.
