# Supplementary material for: Emergence of a barium metal-organic framework for mitigating off-target effects of alpha radionuclide therapy
Source: Theranostics. 2026 Jan 1;16(4):1804–17. doi: 10.7150/thno.121316 (PMC12680533; doi:10.7150/thno.121316)
Supplement: Supplementary file 1 — Supplementary methods, figures and tables. [file thnov16p1804s1.pdf]

Supporting Information

Emergence of a barium metal-organic framework for mitigating off-target effects of alpha radionuclide therapy

*Long Qiu[1,2,3], Jie Lyu[1], Yuqi Guo[1], Shilong Shi[1], Xijian Chen[1,4], Junshan Geng[3], Qian Xiao[3], Jiali Liao[1], Yuanyou Yang[1], Jinsong Zhang[3], Ning Liu[1], Feize Li[1]**

[1] Key Laboratory of Radiation Physics and Technology of the Ministry of Education, Institute of Nuclear Science and Technology, Sichuan University, Chengdu 610064, P. R. China

[2] Department of Nuclear Medicine, Chongqing University Cancer Hospital, Chongqing 400030, P. R. China

[3] Sichuan Engineering Research Center for Radioactive Isotope, National Engineering Research Center for Isotopes and Pharmaceuticals, Nuclear Power Institute of China, Chengdu 610005, P. R. China

[4] Department of Nuclear Medicine, West China Hospital of Sichuan University, Chengdu, Sichuan 610041, P. R. China.

Table of contents

S1. Methods..........3

1.1. Characterizations..........3

1.2. XPS, XAS, and TEM Analysis of M^{n+}@AEMOF-6..........3

1.3. *In vitro* Stability Characterization..........5

1.4. Cellular Experiments..........5

S2. Supplementary Figures..........8

S3. Supplementary Tables..........16

S1. Methods

1.1. Characterizations

The crystalline information of the examined samples was analyzed using a powder X-ray diffractometer (PXRD, Bruker D8 Advance diffractometer). Fourier transform infrared spectrometry (FTIR) spectra were collected using a NICOLET iS50 FT-IR spectrometer in the range of 500 to 4000 cm^{-1}. The micromorphology of relevant samples was determined using high-resolution transmission electron microscope (TEM, JEM-F200). The hydrodynamic size and zeta potential of the nanoparticles were measured using a dynamic light scattering instrument (DLS, Malvern Zetasizer Nano ZS90). An HPGe detector with GammaVision software and a CRC-15 dose calibrator were adopted to determine the radioactivity of the prepared ^{89}Zr and ^{224}Ra-radiolabeled compounds without decay correction.

1.2. XPS, XAS, and TEM Analysis of M^{n+}@AEMOF-6

To minimize the handling of radioactive radionuclides and avoid the generation of radioactive waste solutions during the adsorption experiments, naturally isotopes of Tl, Bi and Pb were used as surrogates of the radioactive ^{208}Tl, ^{212}Bi, and ^{212}Pb, respectively. Initially, the adsorption of AEMOF-6 for Tl, Bi and Pb ions was examined through a batch adsorption method. These experiments were conducted in 100 mL polypropylene bottles, which were shaken on an oscillator (NTS-4000C, TOKYO RIKAKIKAI) at 90 strokes per min at room temperature. Specifically, 50 mg of AEMOF-6 nanoparticles were introduced into bottles containing 50 mL solutions of Tl, Bi, and Pb ions, respectively, at a concentration of 20 ppm. Upon completion of the adsorption

experiments, the M^{n+}@AEMOF-6 particles were centrifuged, washed with water, and subsequently dried in an oven. After that, the chemical composition of the samples was analyzed by XPS.

The Pb@AEMOF-6 and Bi@AEMOF-6 XAS spectra were collected at beamline BL13SSW of Shanghai Synchrotron Radiation Facility in China at room temperature, with extended X-ray absorption fine structure (EXAFS) spectra recorded in transmission or fluorescence mode. Data fitting was carried out using the Athena and Artemis programs.

The morphology of AEMOF-6 loaded with Pb, Bi, and Tl, *i.e.*, Pb@AEMOF-6, Bi@AEMOF-6, and Tl@AEMOF-6 were analyzed by TEM. Based on the highest administered dose of 55.5 kBq ^{224}Ra used in the animal experiments for mice bearing tumors of $\leqslant$1000 mm^3, the calculated concentrations of ^{212}Pb, ^{212}Bi, and ^{208}Tl are 1.08×10^{-3}, 1.02×10^{-4}, and 5.07×10^{-6} μg/L (ppb), respectively. We 1000-fold scaled up the concentrations of these daughter radionuclides to 1.08, 1.02×10^{-1}, and 5.07×10^{-3} ppb for ^{212}Pb, ^{212}Bi, and ^{208}Tl, respectively, relating to 55.5 MBq ^{224}Ra in potential clinical applications. To minimize the handling of radionuclides and avoid generating radioactive wastes during adsorption experiments, naturally isotopes of Tl, Bi, and Pb were used as surrogates for ^{208}Tl, ^{212}Bi, and ^{212}Pb during adsorption experiments, respectively. AEMOF-6 nanoparticles were placed into the solutions containing these cations for 12 h, then the obtained Pb@AEMOF-6, Bi@AEMOF-6, and Tl@AEMOF-6 were centrifuged and washed with water. After dried in an oven, the micromorphology of AEMOF-6 loaded with different cations was analyzed by TEM.

1.3. *In vitro* Stability Characterization

For the evaluation of *in vitro* stability, ^{224}Ra-AEMOF-6 and ^{224}Ra-AEMOF-6@CS were incubated and shaken on an oscillator at 90 strokes per min at room temperature in both 7 mL of saline solution and 20% fetal bovine serum (FBS) in phosphate-buffered saline (PBS). The radiochemical stability of ^{224}Ra-AEMOF-6 and ^{224}Ra-AEMOF-6@CS was assessed by measuring the radioactivity in the supernatant and the precipitate after centrifugation at 13000 rpm for 10 min using an FH463B γ well-type scintillation intelligent detector (China National Nuclear Corporation, Beijing, China).

1.4. Cellular Experiments

1.4.1. Cell Culture and Treatment

Murine breast cancer (4T1) cells were obtained from American Type Culture Collection (ATCC). Typically, 4T1 cells were dislodged from the liquid nitrogen tank and promptly immersed in a 37 °C water bath for rapid thawing, after which the thawed cells were transferred to a centrifuge tube containing 4.0 mL of culture medium and centrifuged at 1000 rpm for 4 min to remove residual dimethyl sulfoxide (DMSO) from the cryopreservation solution. Finally, a fresh culture medium was added, and the cells were gently resuspended by pipetting before being transferred to a culture flask. The 4T1 cells were then maintained in Roswell Park Memorial Institute 1640 (RPMI-1640) cell culture medium containing 10% FBS and 1.0% penicillin−streptomycin, inside a humidified incubator (37 °C, 5% CO_2). After the 4T1 cells were cultured to 80% density the original culture medium was removed, and the adhered cells were

rinsed with fresh PBS to flush away the residua culture medium. Trypsin (2.0 mL) was added to the culture flask and placed under the inverted microscope. When cell detachment was observed, the culture medium was added to terminate the digestion. The cell-trypsin mixture was transferred to a centrifuge tube and centrifuged at 800 rpm for 4 min. The resulting cell pellets were resuspended in the fresh culture medium by gentle pipetting and then transferred to the new culture flask for further incubation.

1.4.2. Cellular Uptake Experiments

For the cellular uptake assay, the 4T1 cells were seeded in 6-well plates for 24 h. The medium was replaced by RMPI-1640 without FBS for 1 h before the radioligand was added. Then, the cells were incubated with 1 kBq ^{89}Zr, ^{89}Zr-AEMOF-6, and ^{89}Zr-AEMOF-6@CS for 1, 3, 6, 12, and 24 h. After washing and lysis, the cell binding rates of the radiotracers were obtained. The radioactivity was measured using a γ-counter and calculated as the percentage of the applied dose.

1.4.3. Cellular Internalization Experiments

As for internalization experiments, the surface-bound activity was removed by incubating the cells with 1 mL of a glycine-HCl buffer for 15 min. The radioactivity of internalization was evaluated at different incubation time points. The radioactivity was measured using a γ-counter and calculated as the percentage of the applied dose.

1.4.4. Cellular Cytotoxicity Experiments

The cytotoxicity was determined by cell counting kit-8 (CCK8) assay. The 4T1 cells were inoculated into 96-well plates (1000 cells per well) and then treated with free ^{224}Ra, AEMOF-6,

and ^{224}Ra-AEMOF-6@CS at various concentrations. A total of 10 μL of CCK-8 solution and 90 μL of fresh RPMI-1640 were added into each well. Finally, the absorbance at 450 nm was measured using a multifunctional enzyme marker.

S2. Supplementary Figures

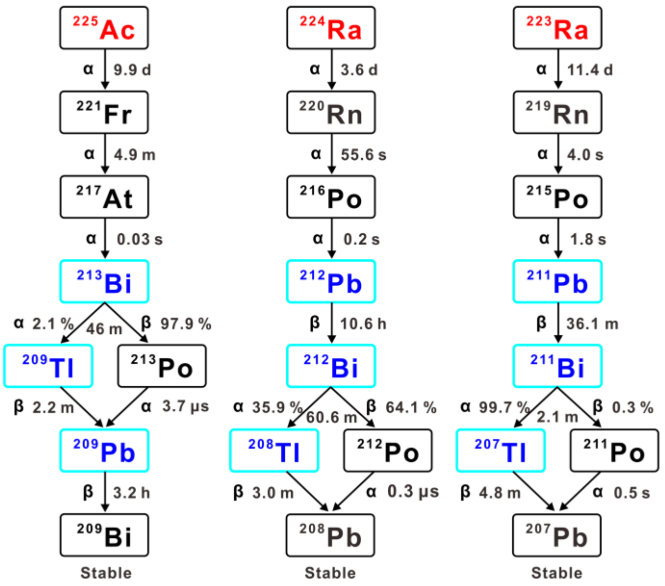

Figure S1. Decay chain of ^{225}Ac, ^{224}Ra, and ^{223}Ra.

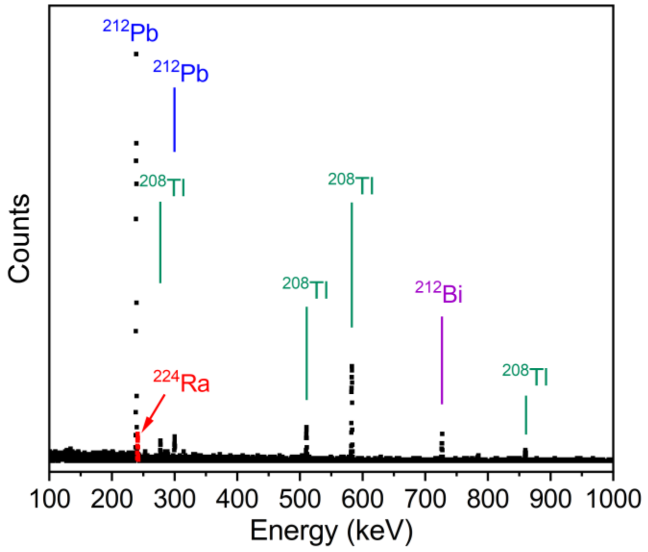

Figure S2. The γ-energy spectrum diagram of ^{224}Ra-containing solution. Data points corresponding to the characteristic γ-peak of ^{224}Ra within the energy range of 240.2~241.8 keV are highlighted in red for better visibility.

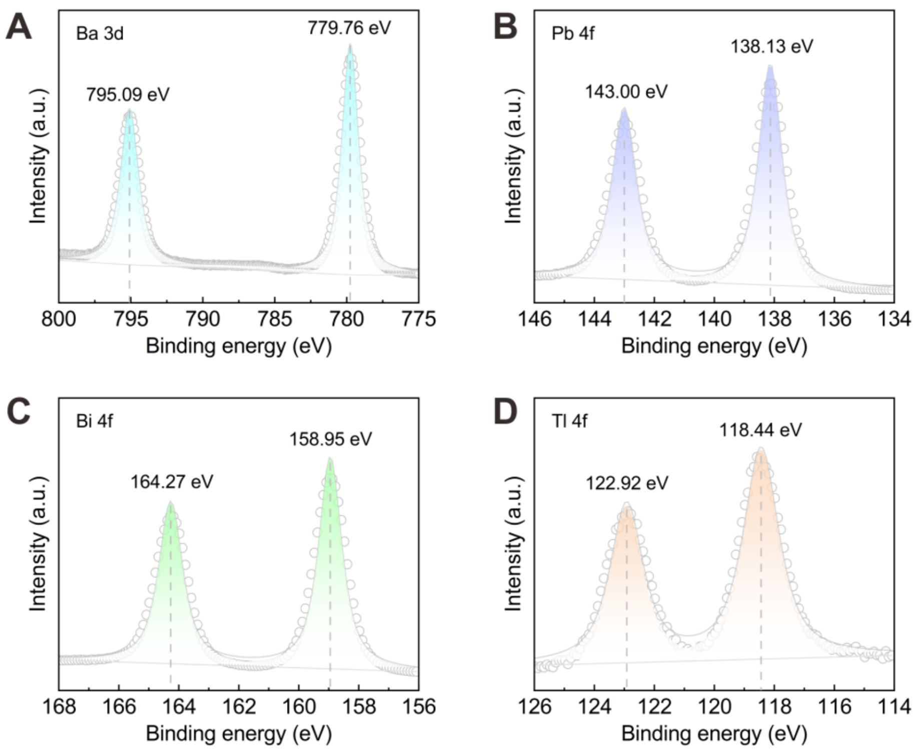

Figure S3. XPS (A) Ba3d high-resolution spectra of AEMOF, (B) Pb4f high-resolution spectra of Pb@AEMOF, (C) Bi4f high-resolution spectra of Bi@AEMOF, and (D) Tl4f high-resolution spectra of Tl@AEMOF. The results indicate that the oxidation states of Ba, Pb, Bi, and Tl are +2, +2, +3 and +1 in AEMOF, Pb@AEMOF, Bi@AEMOF and Tl@AEMOF, respectively.

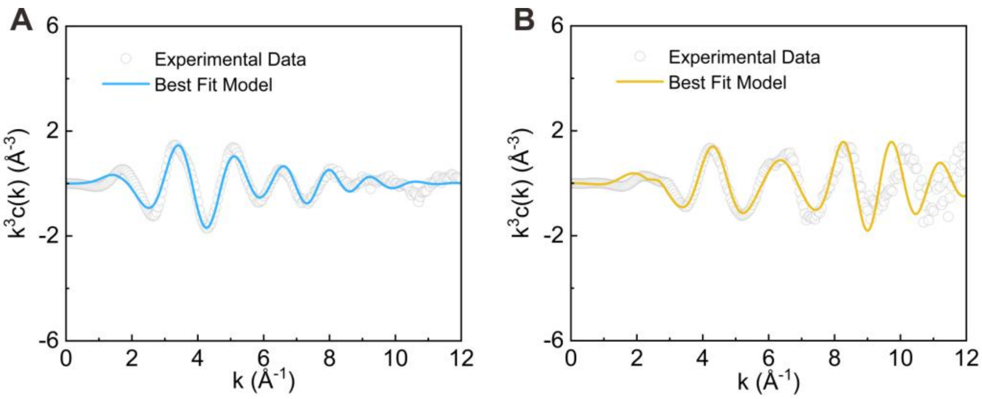

Figure S4. The k-space and best fit for the L_3-edge of (A) Pb@AEMOF-6 and (B) Bi@AEMOF-6. Artemis software analysis revealed 8-coordinate geometries for both metal ions, with average bond distances of 1.79~2.13 Å for Pb-O and 2.24~2.53 Å for Bi-O.

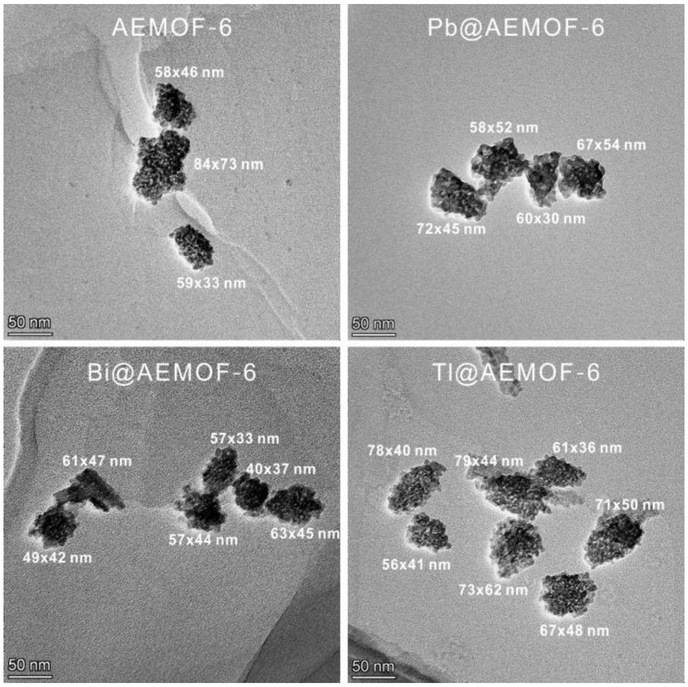

Figure S5. TEM image of AEMOF-6, Pb@AEMOF-6, Bi@AEMOF-6, and Tl@AEMOF-6. AEMOF-6 maintained its block-like micromorphology after loading with Pb, Bi, and Tl, showing good structure stability.

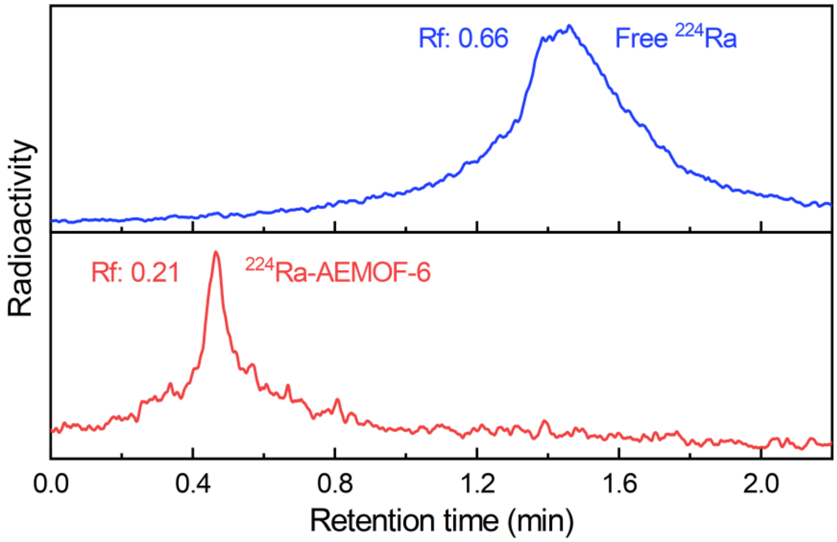

Figure S6. Radio-TLC chromatogram of free ^{224}Ra and ^{224}Ra-AEMOF-6 using 1 mM DTPA as developing agent.

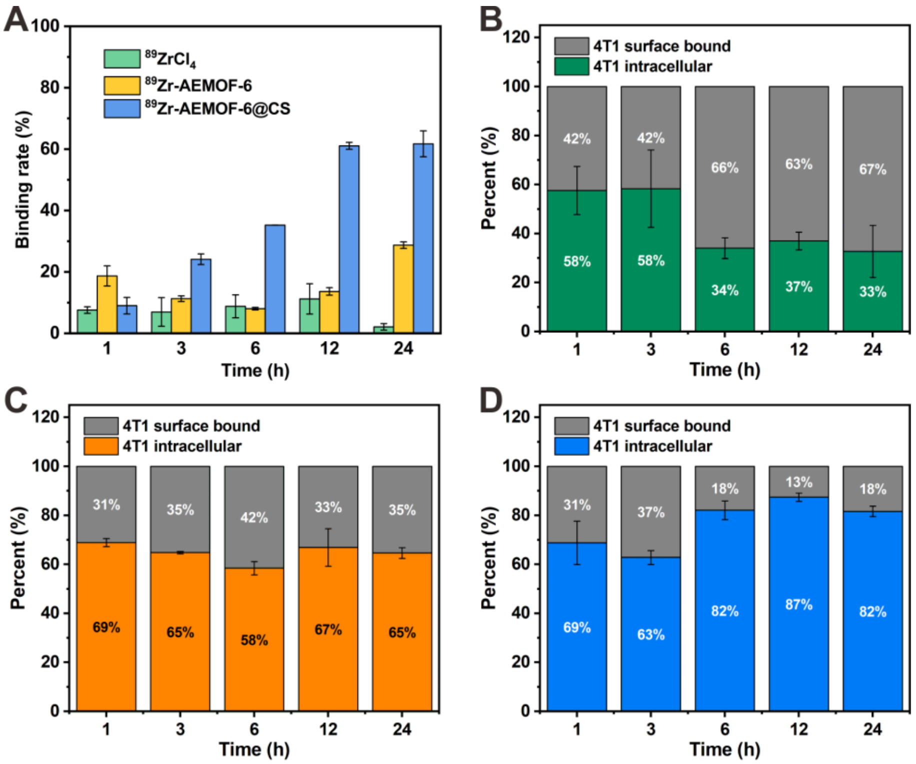

Figure S7. (A) Binding rates of ^{89}Zr, ^{89}Zr-AEMOF-6, and ^{89}Zr-AEMOF-6@CS to 4T1 cells at different timepoints. Internalization of (B) ^{89}Zr, (C) ^{89}Zr-AEMOF-6, and (D) ^{89}Zr-AEMOF-6@CS into 4T1 cells after incubation for 1, 3, 6, 12, and 24 h. AEMOF-6@CS exhibits significantly enhanced cancer cell binding and improved internalization ability toward 4T1 cell line. This may be assigned to the biocompatible and protonated surface of AEMOF-6@CS, which is favorable for cancer cell to endocytose corresponding nanoparticles.

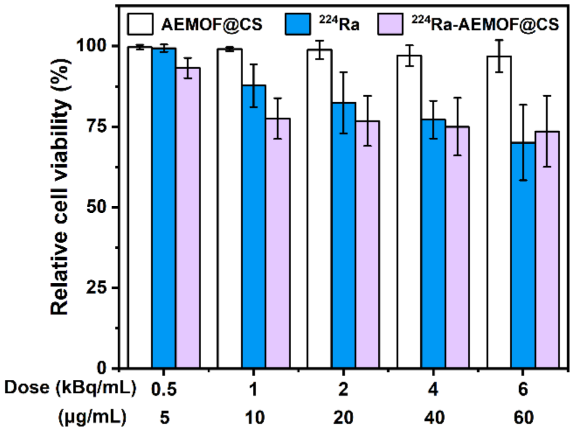

Figure S8. The relative viability of 4T1 cells incubated with AEMOF-6@CS, free ^{224}Ra, and ^{224}Ra-AEMOF-6@CS at different concentrations for 12 h. Benefiting from the high cellular binding and internalization efficiency of ^{224}Ra-AEMOF-6@CS, a significantly enhanced inhibitory effect on cell viability was observed in cytotoxicity assays. In contrast, AEMOF-6@CS carrier itself produced no significant cytotoxicity at a wide concentration range (5~60 μg/mL), confirming that the observed cell death was specifically caused by high linear energy transfer (LET) radiation from the decay of ^{224}Ra. These results highlight the therapeutic potential of ^{224}Ra-AEMOF-6@CS as an alpha-particle delivery system.

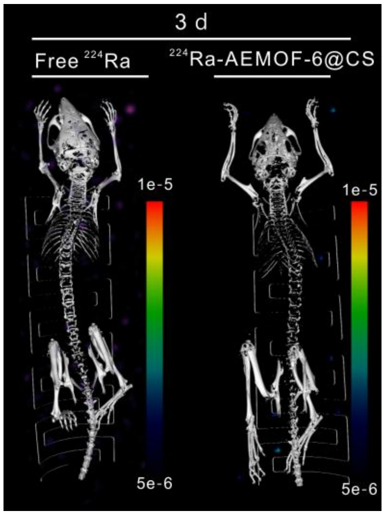

Figure S9. Micro-SPECT/CT imaging in subcutaneous 4T1 tumor-bearing mice after *i.t.* injection of free ^{224}Ra or ^{224}Ra-AEMOF-6@CS for 3 days. At this timepoint, neither the 37.0 kBq free ^{224}Ra group nor the ^{224}Ra-AEMOF-6@CS group exhibited adequate SPECT imaging signals.

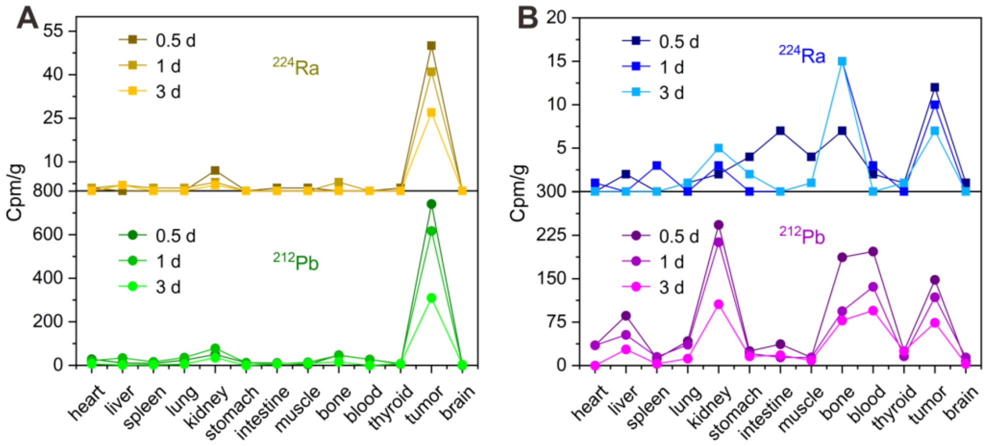

Figure S10. The radioactivity accumulation uptake of ^{224}Ra at 241.0 keV and ^{212}Pb at 238.6 keV of subcutaneous 4T1 tumor-bearing mice after *i.t.* injection of (A) ^{224}Ra-AEMOF-6@CS and (B) free ^{224}Ra for 0.5, 1, and 3 days. In the ^{224}Ra-AEMOF@CS group, both ^{224}Ra and ^{212}Pb were predominantly localized in the tumor site. In contrast, in the free ^{224}Ra group, ^{224}Ra was mainly accumulated in the bone and tumor, while ^{212}Pb was widely distributed in the kidney, bone, blood, and tumor, indicating significant diffusion of the daughter radionuclides.

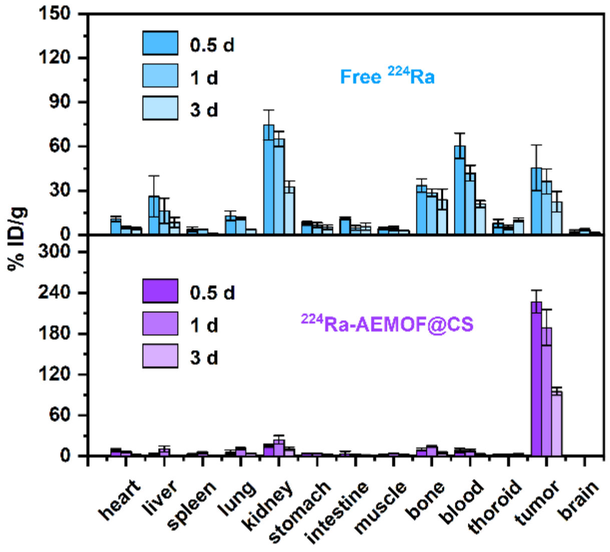

Figure S11. The biodistribution of free ^{224}Ra and ^{224}Ra-AEMOF-6@CS after *i.t.* injection at 0.5, 1, and 3 d *p.i.* Compared to the free ^{224}Ra group, significantly lower radioactivity uptake was detected in the blood, liver, kidney, lung, and bone of the group treated with ^{224}Ra-AEMOF-6@CS, indicating enhanced *in vivo* radioactivity localization capability.

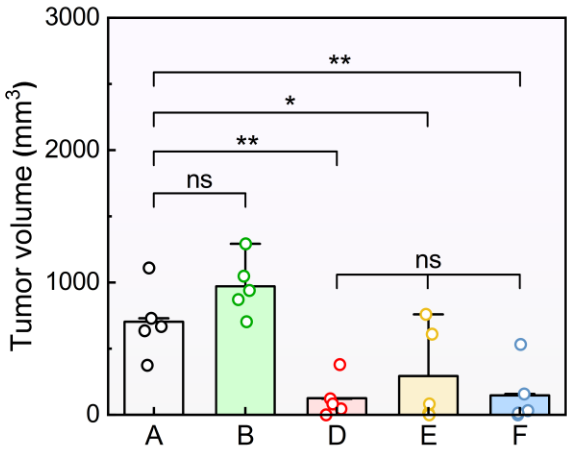

Figure S12. Statistical analysis of tumor volume change in the treatment groups [(A) Saline, (B) AEMOF-6@CS, (D) 18.5 kBq ^{224}Ra-AEMOF-6@CS, (E) 37.0 kBq ^{224}Ra-AEMOF-6@CS, and (F) 55.5 kBq ^{224}Ra-AEMOF-6@CS] on day 21, using t-test. *P < 0.05, **P < 0.01, ***P < 0.001, ****P < 0.0001.

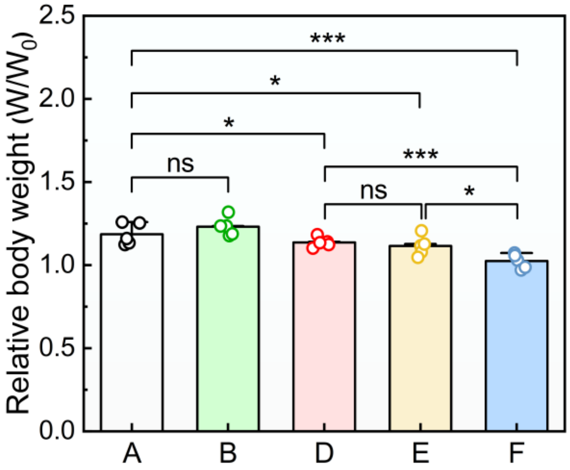

Figure S13. Statistical analysis of relative body weight change in the treatment groups [(A) Saline, (B) AEMOF-6@CS, (D) 18.5 kBq ^{224}Ra-AEMOF-6@CS, (E) 37.0 kBq ^{224}Ra-AEMOF-6@CS, and (F) 55.5 kBq ^{224}Ra-AEMOF-6@CS] on day 21, using t-test. *P < 0.05, **P < 0.01, ***P < 0.001, ****P < 0.0001.

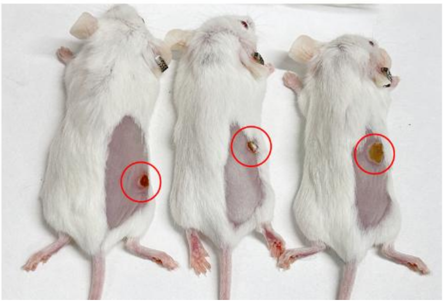

Figure S14. The optical photos of the 37.0 kBq free ^{224}Ra group at 3 d *p.i.* Severe skin ulceration was observed in these animals, necessitating immediate euthanasia.

S3. Supplementary Tables

Table S1. The changes of bing energy (ΔE) of $Ba(DHB)_6$ associated with the coordination process.

Name	$(DHB)_6$	Ba	$Ba(DHB)_6$
Structures	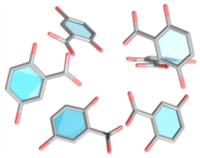	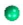	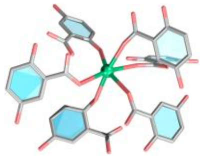
E (Hartrees)	-3420.050858	-25.395013	-3447.654029
ΔE (Hartrees)		-2.208158	
ΔE (kJ/mol)		-5797.519271	

Table S2. The changes of bing energy (ΔE) of $Ra(DHB)_6$ associated with the coordination process.

Name	$(DHB)_6$	Ra	$Ra(DHB)_6$
Structures	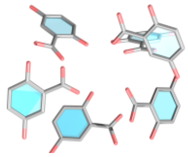	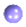	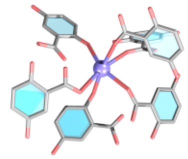
E (Hartrees)	-3420.054150	-23.987159	-3446.223999
ΔE (Hartrees)		-2.18269	
ΔE (kJ/mol)		-5730.65303	

Table S3. The EXAFS fit results for Pb foil, Pb@AEMOF-6, Bi foil, and Bi@AEMOF-6 (*R* is the interatomic distance, N is the coordination number, σ^2 is the Debye-Waller Factor, ΔE_0 is the energy threshold value).

Sample	Scattering path	N	*R*	σ^2	ΔE_0	R factor	S_0
Pb foil	Pb-Pb	12	3.41±0.01	0.0290	-6.02±0.74	0.0006	0.90
Pb@AEMOF-6	Pb-O	4	1.79±0.01	0.0146	-9.90±0.01	0.0010	0.90
	Pb-O	4	2.13±0.01	0.0150			
	Pb-C	6	2.98±0.01	0.0003			
Bi foil	Bi-Bi	6	3.08±0.01	0.0096	-8.77±1.49	0.0067	0.95
	Bi-Bi	6	3.37±0.01	0.0143			
Bi@AEMOF-6	Bi-O	4	2.24±0.01	0.0099	2.58±0.61	0.0065	0.95
	Bi-O	4	2.53±0.01	0.0077			
	Bi-C	6	2.88±0.01	0.0143			

Table S4. The radioactivity accumulation uptake of ^{224}Ra at 241.0 keV (4.10%), ^{212}Pb at 238.63 keV (43.6%) and ^{212}Bi at 727.33 keV (6.67%) in tumor, blood, liver, kidney, and bone of subcutaneous 4T1 tumor-bearing mice after *i.t.* injection of free ^{224}Ra on 0.5, 1, and 3 d.

Organ or Tissue	Radioactivity accumulation of free ^{224}Ra (cpm/g)								
	0.5 d			1 d			3 d		
	^{224}Ra	^{212}Pb	^{212}Bi	^{224}Ra	^{212}Pb	^{212}Bi	^{224}Ra	^{212}Pb	^{212}Bi
Tumor	12	148	10	10	118	12	7	74	15
Blood	1	197	27	3	136	14	1	95	7
Liver	0	86	4	0	54	11	0	28	11
Kidney	0	244	11	1	213	21	2	106	11
Bone	1	187	2	2	94	0	2	78	2

Table S5. The radioactivity accumulation uptake of ^{224}Ra at 241.0 keV (4.10%), ^{212}Pb at 238.63 keV (43.6%) and ^{212}Bi at 727.33 keV (6.67%) in tumor, blood, liver, kidney, and bone of subcutaneous 4T1 tumor-bearing mice after *i.t.* injection of ^{224}Ra-AEMOF-6@CS on 0.5, 1, and 3 d.

Organ or Tissue	Radioactivity accumulation of ^{224}Ra-AEMOF-6@CS (cpm/g)								
	0.5 d			1 d			3 d		
	^{224}Ra	^{212}Pb	^{212}Bi	^{224}Ra	^{212}Pb	^{212}Bi	^{224}Ra	^{212}Pb	^{212}Bi
Tumor	49	742	49	41	617	22	27	310	20
Blood	0	27	4	0	27	0	0	0	0
Liver	0	11	2	0	34	2	0	0	0
Kidney	1	28	2	2	53	0	1	23	0
Bone	0	31	1	2	36	2	0	15	0
